# Supplementary material for: Higher Prevalence of Nonsense Pathogenic DMD Variants in a Single-Center Cohort from Brazil: A Genetic Profile Study That May Guide the Choice of Disease-Modifying Treatments
Source: Brain Sci. 2023 Oct 28;13(11):1521. doi: 10.3390/brainsci13111521 (PMC10669865; doi:10.3390/brainsci13111521)
Supplement: Supplementary file 1 [file brainsci-13-01521-s001.zip › brainsci-2625002-supplementary.pdf]

Supplementary Table S1. Profile of point Mutations in Northeast brazilians.

Mutation	Patients	Nucleotide change	Protein change	Phenotype
Nonsense	4	c.8038 C>T	p.Arg2680*	DMD
Nonsense	3	c.453 T>G	p.Try151*	DMD
Nonsense	2	c.433 C>T	p.Arg145*	DMD
Nonsense	2	c.6283 C>T	p.Arg2095*	DMD
Splice-site	2	c.3603+A>T		DMD
Splice-site	2	c.3603+3A>T		BMD/DMD
Nonsense	1	c.3151C>T	p.Arg1051*	DMD
Frameshift	1	c.4314_4315delAA CTT >	p.Arg1439Serfs*6	DMD
Nonsense	1	c.6292 C>T	p.Arg2098*	DMD
Nonsense	1	c.8608C>T	p.Arg2870*	DMD
Nonsense	1	c.8945 G > A	p.Arg2982*	DMD
Nonsense	1	c.9337C>T	p.Arg3113*	DMD
Frameshift	1	c.141dupG	p.Arg48Glufs*41	DMD
Frameshift	1	c.2552_2553insA G>GT	p.Asn851Lysfs*17	DMD
Nonsense	1	c.10011C>A	p.Cys3337*	DMD
Frameshift	1	c.3295_3296delCA CTG>C	p.Gln1099Asp fs*11	DMD
Frameshift	1	c.5131delC TG > T	p.Gln1711Serfs*10	DMD
Nonsense	1	c.Gln3037C>T	p.Gln3037*	DMD
Nonsense	1	c.133C>T	p.Gln45*	DMD
Frameshift	1	c.3533_3536delAAGA	p.Glu1178Glyfs*22	DMD
Frameshift	1	c.9269_9270delAG GCT>G	p.Glu3090Alafs*	DMD
Frameshift	1		p.Glu701Arg fs*28	DMD
Frameshift	1	c.3185_3192delinsTTT GTAT	p.Lys1062Ilefs*10	DMD
Frameshift	1	c.3396delA	p.Lys1132Asnfs*20	DMD
Frameshift	1	c.6974delA - CCDS5S395.1	p.Lys2325Serfs*	DMD
Nonsense	1	c.8744 G>A	p.Trp2915*	DMD
Nonsense	1	c.9248G>A	p.Trp3083*	DMD
Nonsense	1	c.5646 C>A	p.Tyr1882*	DMD
Nonsense	1	c.6276C>A	p.Tyr2092*	DMD
Splice-site	1	c.5740-1G>T		BMD
Splice-site	1	c.9362-1G>C		DMD
Splice-site	1	c.2804-1del		DMD
Splice-site	1	c.2169-1G>A		DMD
Splice-site	1	c.9286+2delT		DMD
